# A privacy-preserving design for sharing demand-driven patient datasets over permissioned blockchains and P2P secure transfer

**DOI:** 10.7717/peerj-cs.568

**Published:** 2021-06-09

**Authors:** Mercedes Rodriguez-Garcia, Miguel-Angel Sicilia, Juan Manuel Dodero

**Affiliations:** 1 Superior School of Engineering, University of Cádiz, Puerto Real, Cádiz, Spain; 2 University of Alcalá, Alcalá de Henares, Madrid, Spain

**Keywords:** Health information exchange, Data privacy, Blockchain, Smart contract

## Abstract

Sharing patient datasets curated by health institutions is critical for the advance of monitoring, surveillance and research. However, patient data is sensitive data and it can only be released under certain conditions and with previous explicit consent. Privacy preserving data sharing provides techniques to distribute datasets minimizing the risk of identification of patients. However, the sharing of datasets is typically done without considering the needs or requests of data consumers. Blockchain technologies provide an opportunity to gather those requests and share and assemble datasets using privacy-preserving methods as data and requirements on anonymity match. The architecture and design of such a solution is described, assuming an underlying permissioned blockchain network where providers such as healthcare institutions deal with consent, patient preferences and anonymity guarantees, playing a mediator role to a network of organizations.

## Introduction

Information privacy is concerned with the usage, transfer and processing of personal data. Public and private sector organizations may use, transfer or process individuals’ data, thus affecting individual autonomy and the choices we can make. This makes protection of personal data a key concern for regulators, and sharing is restricted to conditions in which explicit consent must be given by individuals, and eventual transfer should also take appropriate security means to avoid leaking or disclosure. Health data as collected and curated in healthcare institutions is sensitive, personal data enriched with the result of evaluations, indications or observations made by professionals. As such, anonymized collection and sharing of healthcare data is of great value to surveillance, monitoring and research, especially for conditions or diseases for which data or knowledge is less available (Emam, Rodgers & Malin, 2015).

Current approaches to sharing healthcare datasets rely upon processes of anonymization using techniques that control the risk of identity disclosure of individuals (Rocher, Hendrickx & Montjoye, 2019), and are not carried out automatically but as the result of occasional or human-initiated processes. This is ignoring the fact that patients may have different risk preferences, which could be combined in the production of datasets, and it also misses opportunities to aggregate datasets of diverse sizes and composition as a result of automated requests. Further, many patients are willing to share data for the sake of improving other’s health, but incentives or rewards may increase the possibilities of voluntary sharing, without the need of a completely commodified market approach.

Commodities represent something that can be reduced to money without changing in value and is interchangeable with every other commodity in terms of exchange value. The commodification of personal information threatens individual privacy and raises a question on free alienability of personal data. The alienability of personal data poses a controversy regarding whether restrictions should be defined on its exchange. The establishment of a trading market of personal information that respects individual privacy involves limitations on an individual’s right to alienate personal information and the definition of rules that force disclosure of the terms of trade (Schwartz, 2004). Concerning personal healthcare data, their alienability implies that the privacy interest in protecting it should be overridden by significant social needs, such as clinical research or public health reporting (Schwartz, 1997). For example, rapidly reporting an infectious disease outbreak can be a reason to counter-balance the privacy restrictions on individuals’ healthcare data.

Some frameworks for privacy-aware trading of personal data define an explicit and negotiable privacy policy that defines the purpose, type of data, retention period and price (Iyilade & Vassileva, 2013). These rules, expressed as purpose-to-use (P2U) privacy policy specifications, may disclose the terms of trade, but the way to enforce the abidance of such rules is not considered. P2U describes a case of healthcare data trading in which the data owners directly negotiate a compensation for sharing their data. Putting users/owners in control of their own data is the foundation of Personal Data Ecosystem tools and initiatives (Cavoukian, 2012), such as SOLID (Mansour et al., 2016) and, in general, all Privacy by Design (PbD) frameworks. Yet such direct owner-customer interactions are not usual in the healthcare sector, which usually relies on custodians such as hospitals or regional healthcare services to keep and manage owners’ data in their healthcare databases and Electronic Medical Records (EMR). Privacy regulations that restrict custodians to release their patients’ information have led to a significant reduction on the adoption of EMRs (Miller & Tucker, 2009). Besides, individuals’ ability to make informed decisions about their privacy is hindered since they can have imperfect or asymmetric information regarding the purposes and consequences of their data be collected (Acquisti, Taylor & Wagman, 2016).

The economic effects of data markets raise a lot of privacy threats that can be fought through confusion and distraction strategies (Jensen, 2013), which have been long investigated in the privacy-preserving data publishing (PPDP) research area. The idea is to limit the purpose-to-use capabilities of shared data by slightly altering the original dataset, instead of by technically enforcing the fulfilment of a set of predefined policies. The restriction of utility over personal data can be part of the trading, after the data owner selects her intended privacy level and a custodian manages the enforcement of the selected privacy rules in a privacy-aware data market.

Schwartz (2004) described and analyzed networked computing technologies and applications having a consequence on personal data privacy. Schwartz’s analysis was focused on the perils of spyware and adware. Blockchain, however, was not on the roadmap of that study on the perils of commodification of personal data. Blockchain is currently the emerging channel for commodification of all kinds of goods, which can be tokenized, mapped to cryptocurrencies and interchanged by individuals and organizations in open, global markets. As argued before, individual privacy concerns and the alienability of personal data are of great interest, especially in blockchain markets.

Besides traditional institution-driven EMR data interoperability, there is a push towards patient-driven interoperability, in which health data exchange is patient-mediated (Gordon & Catalini, 2018). Blockchain enables patients to retain control over their data. Patient-driven interoperability, however, brings new challenges around security and privacy that must be addressed at scale. Given the proper privacy measures, blockchain can provide a higher degree of anonymity around patient identity than in a centralized system. Blockchain can also manage audit trails while keeping the data private (Theodouli et al., 2018).

The focus of this paper is on the PPDP techniques that can be used to limit the purpose and consequences of sharing personal healthcare datasets using the possibilities of blockchain technologies. Concretely, the contributions of the design described in this paper are the following: (1) providing an auditable mechanism for patients to give consent on the use of their data, (2) giving interested stakeholders the opportunity to express healthcare data needs, (3) a decision mechanism by which healthcare institutions can fairly respond to these needs as consents become available and datasets can be automatically produced, and transfer data in which complete auditing of desired risk levels is possible, and (4) a mechanism to reward users for sharing events, that accounts for their preferences. This design is intended for permissioned blockchain networks in which entities are identified and full data protection regulation enforcement is possible, introducing also built-in neutrality or fairness so that no data requests are disregarded or discriminated. This is an important element in the case of public–private systems in which entities requesting data from public institutions may be competing, or in cases in which it is purposely intended that the intermediaries are not making profit but just facilitating the data exchange.

The rest of this paper is structured as follows. ‘Background on data privacy’ describes privacy models and PPDP techniques to protect personal information when it is shared with third parties. ‘Privacy-preserving medical data sharing model’ describes our privacy-preserving model for sharing medical data over permissioned blockchains and P2P secure transfer, as well as supporting technology. ‘Discussion’ analyzes the benefits of the proposed model. ‘Performance evaluation’ evaluates the proposed model on a permissioned blockchain network. Finally, conclusions and future research issues are summarized in ‘Conclusions and outlook’.

### Background on data privacy

The possibility of using blockchain as a platform to share medical data in the healthcare ecosystem (e.g., among patients, hospitals, insurance companies, pharmaceutical companies and research institutes) has recently received considerable attention (Azaria et al., 2016; Dubovitskaya et al., 2018; Liang et al., 2017). In order to increase interoperability among healthcare providers and to keep an auditable history of transactions, Azaria et al. (2016) proposes sharing medical data on permissionless blockchains. With this proposal, patients can selectively share their health data with data requestors by stating the access permissions through provisions in smart contracts. Other works (Dubovitskaya et al., 2018; Liang et al., 2017) explore the use of permissioned blockchains to maintain an access control layer that allows certain actions to be performed only by certain identifiable participants. The membership service supported by the permissioned blockchain is responsible for member enrollment and generating access control lists according to patients’ requirements. By establishing access permissions in the transaction certificates issued by the membership service, patients can selectively share their health data with the data requestors. All these proposals provide the patients with a possibility to specify access control over their data, however, none of them addresses de-identification of sensitive information in accordance with data protection regulations.

Different regulations on data protection have been adopted to preserve individuals’ privacy, such as the European Union General Data Protection Regulation (European Parliament, 2016) that regulates the protection of personal data in any field of activity or the United States Health Insurance Portability and Accountability Act (HIPAA, 2012) intended for the health field. In the specific case of healthcare, data privacy standards focus their application on the so-called *Protected Health Information* (PHI), that is, health data associated with identifying information of the patients. Identifying information includes not only direct identifiers, such as SSN or name, but also any *a priori* non-identifying data that, in combination with other non-identifying data from the dataset, may make a record distinguishable. Findings published in Golle (2006) showed that the 63% of the United States population could be uniquely identified by a combination of simple demographics, such as {gender, ZIP code, birth date}. These pseudo-identifying combinations, named *quasi-identifiers*, may be used by attackers to indirectly identify individuals by linking the dataset with other data sources through a common quasi-identifier (Ciriani et al., 2007; Samarati, 2001). If the exploited external sources contained some direct identifier, attackers could determine the identity of the individuals recorded in the dataset. The *linking attack* (or *re-identification attack*) constitutes a real and serious privacy threat that allows data brokers to compile and aggregate individuals’ data gathered from different sources to construct user profiles (Ramírez et al., 2014). Nowadays, the amount of identifying information externally available in a variety of sources (e.g., voter registration, census data or social media) together with increasing computational power makes it easier to carry out such re-identifications.

Since health information is an especially valuable resource to carry out a variety of studies, the HIPAA Privacy Rule permits a covered entity (i.e., healthcare providers, healthcare clearinghouses or health plans) to share health information without patient consent in either of these two conditions: when the dataset has been *limited* or *de-identified*. To achieve a *limited dataset*, the direct identifiers shown in Table 1 must be removed from the original medical dataset. In this way, none of the remaining attributes in the dataset can be immediately associated with a specific patient. However, despite the dataset has reduced its identifiability, the remaining information still may contain quasi-identifiers that may enable the indirect identification of the patients. For this reason, a limited dataset can only be shared for the purposes of research, public health, or healthcare operations. The second alternative provides a stronger protection on data by also acting on the quasi-identifier attributes from the dataset, either removing them or altering their value. Since the health information in the dataset now ceases to be identifiable, i.e., there is no reasonable basis to believe that the remaining information could be used to identify an individual, this information is no longer PHI and is no longer subject to the Privacy Rule. As a consequence, a *de-identified dataset* can be shared without restrictions. According to the HIPAA Privacy Rule’s de-identification standard (HIPAA, 2012), two methods can be used to yield de-identified health information: *Safe Harbor method* and *statistical method*.

**Table 1:** Information removed in a limited dataset and in a de-identified dataset via Safe Harbor. The difference between both is indicated in italic.

	**Limited dataset**	**De-identified dataset** **(Safe Harbor method)**
**Direct identifiers**	- names	- names
	- postal address information	- postal address information
	- telephone numbers	- telephone numbers
	- fax numbers	- fax numbers
	- email addresses	- email addresses
	- social security numbers	- social security numbers
	- medical record numbers	- medical record numbers
	- health plan beneficiary numbers	- health plan beneficiary numbers
	- account numbers	- account numbers
	- certificate/license numbers	- certificate/license numbers
	- vehicle identifiers	- vehicle identifiers
	- device identifiers	- device identifiers
	- URLs	- URLs
	- IP addresses	- IP addresses
	- biometric identifiers	- biometric identifiers
	- full face photographs	- full face photographs
		- *any other unique identifying number*
**Quasi-identifiers**		- *town, city*
		- *dates (e.g., birth-date)*

The *Safe Harbor method* is based on removing a specific set of direct identifiers and quasi-identifiers from the dataset, as shown Table 1. There is, however, a certain opposition to the medical data disclosing via Safe Harbor because (Malin, Karp & Scheuermann, 2010) (i) the risk of identification may still be significant, since only two quasi-identifier attributes are considered (dates and city), and there may be more quasi-identifier attributes in the dataset that could lead to re-identification (e.g., gender, occupation), and (ii) unlike direct identifiers, quasi-identifier attributes must not be removed from the dataset because they provide useful information for data analysis, e.g., the city is a valuable information for epidemiological studies.

In order to provide provable privacy guarantees and, at the same time, to minimize information loss of the de-identified dataset, the *statistical method* applies a formal protection model to the quasi-identifiers while preserving certain statistics features. One example of a protection model that has been applied to health information is the *k*-anonymity model (El Emam & Dankar, 2008; El Emam et al., 2009). The *k*-anonymity model (Samarati, 2001) is based on homogenizing information to reduce its identifiability. The records in the dataset are homogenized by creating groups of at least *k* records sharing the same values in their quasi-identifier attributes. Since each record in the *k*-anonymous dataset is indistinguishable from at least *k* − 1 other records, the probability of re-identification is limited to 1/*k*. Thus, an attacker with access to identifying data sources containing the same quasi-identifier attributes as the *k*-anonymous dataset will not be able to link the records of a specific patient. At most, the attacker will be able to identify in the de-identified dataset the set of *k* records that contains the target patient. The *k* parameter allows for adjusting the balance between data privacy and integrity and can be used as an indicator of the privacy level. The larger the *k* value, the more anonymous the *k*-anonymous dataset will be. Instead, lower *k* values result in a lesser alteration on data, yielding more useful *k*-anonymous datasets for analysis. Other formal protection models, such as probabilistic *k*-anonymity (Soria-Comas & Domingo-Ferrer, 2012) or ε-differential privacy (Dwork, 2006), can also be considered to de-identify a dataset because, like *k*-anonymity, they enable to set the privacy level that the protected data must satisfy, thereby guaranteeing a minimum level of anonymity to the data subjects. To achieve a stronger protection, additional protection models can be applied to confidential attributes, such as *l*-diversity (Machanavajjhala et al., 2007) or *t*-closeness (Li, Li & Venkatasubramanian, 2007).

These provable privacy guarantees can be attained for a particular dataset using one or several PPDP methods (Fung et al., 2010; Hundepool et al., 2012), as summarized in Table 2. These techniques are based on altering the original values of the quasi-identifier attributes following the guidelines established by a particular protection model while preserving certain statistics features. According to the principle used to alter the quasi-identifiers, we classify these techniques in:

**Table 2:** Privacy-preserving methods used to satisfy protection models.

**Data protection model**	**PPDP method**
*k*-anonymity	- Microaggregation
	- Generalization and local suppression
Probabilistic *k*-Anonymity	- Rank swapping
ε-differential privacy	- Microaggregation and noise addition

- Aggregation-based techniques: the original values are replaced by smaller aggregates, e.g., microaggregation method for numerical (Defays & Anwar, 1998) or nominal (Martínez, Sánchez & Valls, 2013) quasi-identifiers.
- Permutation-based techniques: the original values are swapped, e.g., rank swapping method for numerical (Moore, 1996) or nominal (Rodriguez-Garcia, Batet & Sánchez, 2019) quasi-identifiers.
- Distortion-based techniques: the original values are replaced by their noisy versions, e.g., noise addition method for numerical (Kim, 1986) or nominal (Rodriguez-Garcia, Batet & Sánchez, 2017) quasi-identifiers.
- Abstraction-based techniques: the original values are replaced by more generic values, e.g., generalization method (Samarati, 2001).

Following the particular case of *k*-anonymity, two PPDP techniques can be used to generate *k*-anonymous datasets: generalization and local suppression, and microaggregation. The first one is based on combining the PPDP methods generalization and local suppression (Samarati, 2001). With generalization the values of the quasi-identifier attributes within each group of *k* records are modified by a common category, i.e., a more generic category. Suppression contributes to reduce the amount of generalization required to generate the *k*-anonymous dataset by removing outlier values. This approach has the disadvantage of requiring a high computational cost to find an optimal recoding that minimizes the information loss (Meyerson & Williams, 2004). A second more practical approach is based on microaggregation (Domingo-Ferrer & Torra, 2005). This PPDP method reduces the variability of the attributes to protect by replacing the original values by small aggregates. First, the dataset is partitioned into groups of *k* records following a maximum similarity criterion on the quasi-identifier to protect and, then, the quasi-identifier values in each group are replaced by the group representative value, typically the average value.

It should be noted that there is no particular PPDP method that is universally the best option. Each method has benefits and drawbacks with respect to expected applications of the health information and the features of the dataset (HIPAA, 2012).

### Privacy-preserving medical data sharing model

In this section, we present a new model for sharing medical data that preserves patients’ privacy, their preferences and automatically matches patients’ data with data requests. We first describe the decentralized technologies underlying our model: blockchain networks, InterPlanetary File System, and self-sovereign identity management system. Then, we introduce our privacy-preserving medical data sharing model.

### Blockchain networks

Blockchain is a secure transactions register supported by a peer-to-peer platform (Attili et al., 2016; Kuo, Kim & Ohno-Machado, 2017; Nakamoto, 2008), and in permissioned settings (e.g., using Quorum or Hyperledger Fabric) it can be used to ensure that the parties are guaranteed equal treatment (neutrality). As transactions are generated by the users, these are verified and stored by the network peers, following the established rules by a consensus protocol. Each peer maintains a whole copy of the transactions register, thereby avoiding single-point-of-failure and providing a high level of redundancy. The transactions register is organized in a hash-chain of transactions blocks. This hash-chain interlinks the blocks by embedding in each block the digital fingerprint of the previous block. The digital fingerprint of the previous block is calculated as the cryptographic hash function of all its content, including its embedded digital fingerprint. In this way, if an attacker tampers a transaction in a certain block, the digital fingerprint of that block will change and, as a consequence, the digital fingerprints of subsequent blocks in the chain. This feature not only grants Blockchain an immutable character that allows fraud detection, but also provides a time-stamped native mechanism that facilitates traceability of the operations, since each block can serve as a timestamp of the enclosed transactions.

The decentralized, robustness, immutable and traceable nature of blockchain makes it suitable for applications where independent parties wish to carry out transactions with each other without ceding control to a central management intermediary. One such application is smart contracts, immutable software components that use blockchain, e.g., Ethereum (Wood, 2014), to enforce agreements between contractual parties in the absence of a central management intermediary.

### InterPlanetary File System

InterPlanetary File System (IPFS) (Benet, 2014) is a distributed P2P file system characterized by providing content-addressed file storage. Each file stored in IPFS is identified by its digital fingerprint, which is calculated as a cryptographic hash function of the entire contents of the file. This content identifier is used as a link to access to the file stored in the IPFS platform and as a checksum to detect whether the file content has been tampered or corrupted. Because file storage based on content-addressing grants an immutable character to the stored data, users can trust the content they receive without needing to trust the peers of those who receive it. While IPFS refers to a global, open network of nodes, the same open source software can be used to deploy private IPFS networks with the same functionality. It should be noted that in such a private network, the nodes may be configured or tailored to discard some files, which is important for privacy preserving applications, in which files need to be subject to some sort of “forgetting” about some data, which is difficult to achieve in open, decentralized global networks.

### Self-sovereign identity management system

Self-sovereign identity (SSI) is a model for managing identities that, unlike other approaches, gives identity holders custody and full access control on their credentials. SSI solves security, privacy, and trust problems for existing centralized and federated identity models. This identity management system is based on the Verifiable Credentials Data Model (Sporny, Longley & Chadwick, 2019) and must be deployed on a ledger envisioned to support SSI (hereinafter referred to as SSI ledger), such as that of the framework *Hyperledger Indy*, *Aries* and *Ursa* (Hyperledger Foundation, 2019). *Hyperledger Indy* is a public, permissioned ledger used for the verification of credentials, the component *Aries* provides a set of capabilities to interchange identity proofs between entities following the RFC 0160 - connection protocol (West et al., 2019), and *Ursa* provides the cryptographic implementations necessary to prove and verify the credentials.

*Verifiable credentials* are cryptographically trustworthy attestations about the qualification of an entity (i.e., individual, organization or thing) signed by a trusted third party, e.g., government agencies. These credentials consist of one or more pieces of identity-related information, named *claims*, typically personally identifiable information. To prove identity, entities can generate verifiable presentations of their credentials by including their signature. For example, if an organization is required to prove its legal existence in a contract, it could present the countersigned verifiable credential of its certificate of incorporation, which contains several claims, e.g., the corporation name, its registered number, the date of formation, the type of corporation and the address of its registered office. The signatures contained in a verifiable presentation, i.e., the credential issuer’s signature and the credential owner’s signature, require to be checked with the public verification keys associated with those private keys that were used by the signatories. To eliminate centralized key management authorities and, thus, empower entities to manage their own verification and signing keys, a decentralized public key infrastructure (DPKI) with each entity acting as its own root authority is deployed in the SSI model. The core of this infrastructure is based on so-called decentralized identifiers (DID) (Reed et al., 2020). These identifiers generated by the entities of the SSI ecosystem are globally resolvable to public verification keys and service endpoints, information registered in DID Documents (DDO) that is necessary to verify the signatures of the owner entities and engage in interactions with them. To preserve the privacy of the identity owners, no private credentials are stored on the ledger. Instead, they are stored in secure, private wallets kept by their holders. Only public data, such as credential definitions and schemas, public DIDs, or revocation registers, are stored on the ledger. Off-ledger, entities can securely exchange verifiable presentations as identity proofs via private communication channels between the agents of their respective wallets. On-ledger, entities can verify the received identity proofs.

In the proposed model below, this kind of combination of secure P2P communication and blockchain infrastructure in the case of the interchange between data custodian and customer concerns data aggregated in datasets, since the custodian acts as a surrogate for the interests of the data owners.

### Proposed model

Three actors are defined in the model: *data owner*, *data customer* and *data custodian*. *Data owner* is an individual that takes part in a data collection process supplying his/her own data. We consider a scenario consisting of multiple data owners, each one generating one record with a common data schema. A data owner could be a patient who is contributing with his/her medical data. *Data customer* is the party that acquires (or buys) the medical data for a specific purpose. A data customer could be, for example, a pharmaceutical company that seeks to acquire medical datasets for research and development of new drugs. Finally, *data custodian* is the party that collects and stores data records of multiple owners. A data custodian could be a hospital that, in the exercise of the healthcare, collects and stores medical data of its patients, eventually curated with the outcomes of healthcare events as evaluations, observations or procedures. In the proposed model, we assume that the data owners have a trust relationship with the data custodian, which is legally enforceable and, thus, do not require remaining anonymous against the data custodian.

To carry out data exchange agreements between custodians and customers without central management intermediaries, and that, in turn, these agreements are auditable, irreversible and tamper-proof, these must be made enforceable through smart contracts. Unlike a traditional contract, the agreed-upon conditions in a smart contract are executed in strict compliance by a computer program in the absence of intermediaries. Trust is built around the platform that records and executes the terms of the agreement. Due to decentralized, robustness, immutable and traceable nature of blockchain, this becomes the reference platform for all those applications that, like smart contracts, need to execute trusted transactions between untrusted parties without ceding control to a central party. Thus, we take advantage of these benefits in our model by executing smart contracts on a blockchain for the data request, offer and payment process.

**Figure 1:**
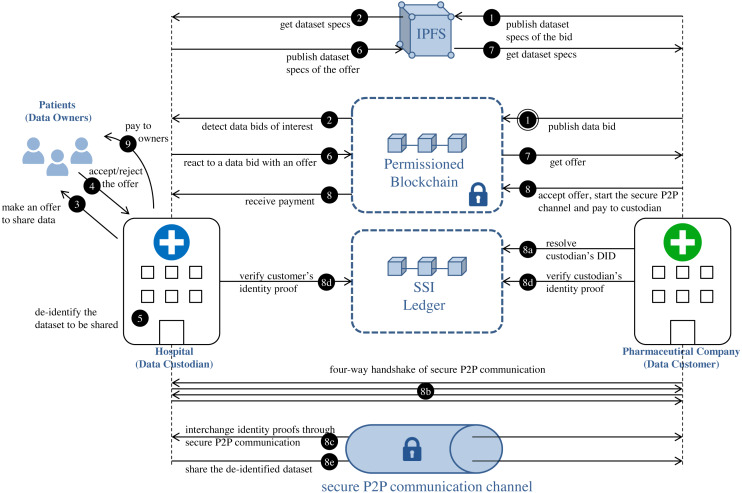
Privacy-preserving medical dataset sharing model. Medical data requirement: steps 1–2. Patients’ authorization collection: steps 3–4. Medical dataset de-identification: step 5. De-identified dataset interchange: steps 6–9.

Figure 1 reflects the different processes that are taking place in the proposed model. This approach decouples the processes at each side of the data custodian system, and the custodian becomes a mediator between the patients and the data customers, creating thus a potential as a “market maker”. The interchange of de-identified medical datasets is carried out via a combination of:

- Smart contracts for the request, offer and payment process.
- Secure P2P communication channels for the actual transfer of anonymized datasets.

In subsequent sections, we describe in detail the medical dataset, as well as the different phases of our model: medical data requirement, patients’ authorization collection, medical dataset de-identification and de-identified dataset interchange.

### Medical dataset

**Figure 2:**
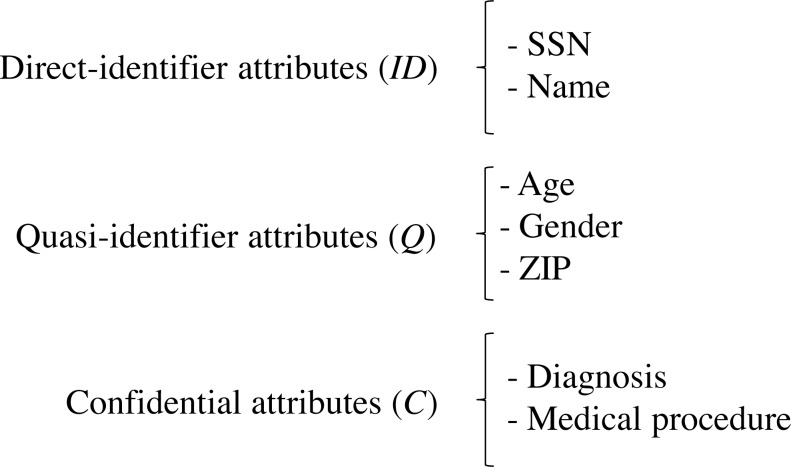
Schema of an example medical dataset.

Let *S* be the data schema of a medical dataset held by the data custodian. *S* is composed of the sets of attributes (*ID*, *Q*, *C*), such that *ID* is the set of direct identifiers, *Q* is the set of quasi-identifier attributes and *C* is the set of confidential attributes. *Q* comprises all the attributes of the data scheme that can potentially be used in record re-identification, which depends on the external information available for the attacker (Soria-Comas & Domingo-Ferrer, 2012), and *C* comprises all the attributes of the data scheme that contains sensitive information on the patients, e.g., patient’s diagnosis. Figure 2 depicts the schema of an example medical dataset.

**Figure 3:**
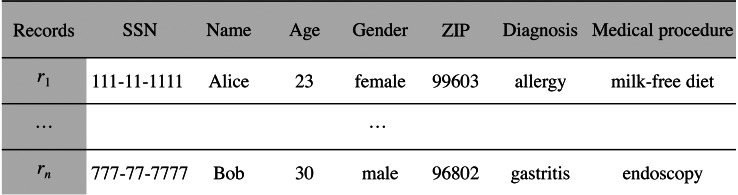
Example medical dataset of *n* records.

Let *D* be the medical dataset held by the data custodian that follows the data schema *S*. *D* can be represented as a table (matrix), where each record (row) contains information about a single patient, and each attribute (column) contains information regarding one of the features collected, such that $D=(ID,Q,C)_{i=1}^{n}$, *n* being the number of records in *D*. We use ${r}_{i}={ \left( ID,Q,C \right) }_{i}$ to refer to the record contributed by the patient *i*. Figure 3 depicts an example medical dataset of *n* records following the schema of Fig. 2.

### Medical data requirement

Data customers can express the need for datasets by publishing data bids on blockchain (step 1 in Fig. 1) through the execution of the function register of the smart contract DatasetBidRegistry (https://github.com/msicilia/pp-datasets-sharing). Basically, a bid contains the customer’s data request, that is: the *expiry date* of the bid, the *dataset specs*, the *intended price* which is the amount willing to pay for the dataset, and a set of *tags* or keywords, such as medical terms from the structured clinical vocabulary SNOMED (Spackman, 2004), to enable efficient searches of the bid. The specs of the dataset report on the structure of the de-identified dataset, and its analytical utility and/or its privacy conditions.

**Figure 4:**
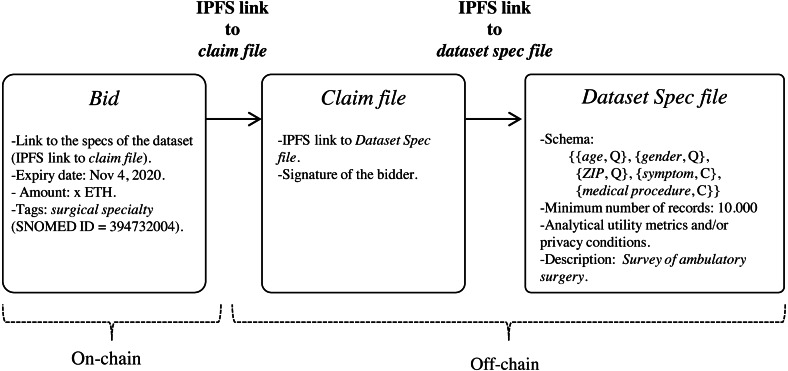
Sequence of IPFS links that must be followed to access the specs of the dataset defined in the bid.

Since the specs of the dataset may be verbose, we propose that they be stored off-chain in a trusted and high throughput platform that makes it accessible to the public, such as IPFS. In this way, data customers will avoid paying high fee for storing significant volumes of data on-chain. With IPFS, only the hash of the file containing the dataset specs needs to be stored on the blockchain. Because IPFS is a content-addressed file storage system, data custodians will be able to use the IPFS hash as a link to access the dataset spec file on IPFS and as a checksum to detect whether the dataset specs have been tampered with. IPFS also provides other benefits such as de-duplication and high availability. Additionally, to certify that the specs of the dataset have been issued by the data customer, thereby avoiding attacks of impersonation of identity, we propose to create an authentication proof that includes the IPFS link to *dataset spec file* signed by the data customer with the private key of its digital certificate (issued by a Certification Authority). This authentication proof is organized in a file, named *claim file*, which is also stored in IPFS. Thus, to access the specs of the dataset, a sequence of IPFS links must be followed, as shown in Fig. 4. The division of the data in a claim file and a dataset spec file separates the concerns of commitment by the data customer (claim) and the descriptions that may be reused (data spec) even by different partners.

Data custodians can detect data bids of interest by executing the function bidinfo of the smart contract DatasetBidRegistry. The tags included in the bids make it easier for data custodian agents to detect which data bids are candidates to be serviced. Local agents in custodians can be set with tags of interest. In this way, all those bids on blockchain that have a term in the field *tag* that matches or is a specialization (according to the hierarchical organization of terms of SNOMED) of any tag configured in the local agent of the custodian will be candidates to be serviced (step 2 in Fig. 1).

This will then be complemented to become scalable as a service by using the decentralized Graph index (https://thegraph.com/). The Graph Index is a network devised specifically for the problems of scalability that we face in this project. While blockchains and storage networks are critical components of any decentralized application, data in them is rarely stored in a format that can be consumed directly in applications. Applications need to filter, sort, paginate, group, and join data before it can be fetched. By using The Graph, developers can query a peer-to-peer network of indexing nodes using GraphQL and verify the results on the client. Currently, The Graph relies on smart contract events, so that the only design requirement imposed by their use is logging as events all the relevant events happening.

### Patients’ authorization collection

In patients’ authorization collection, data custodian make offers to particular patients to share their data based on bids received by potential data customers. This phase must be understood as a process of communication and information between the data custodian and the data owner, a process that ends with the eventual acceptance by the data owner of the sharing of his/her medical data, under some conditions that can be understood as the patient’s preferences. For that, firstly, the custodian proposes to the data owners from the dataset *D* to share their health data under certain privacy and economic conditions (step 3 in Fig. 1). Privacy conditions specify the privacy level that will be applied to the dataset. As an example, if the data protection model considered by the data custodian is *k*-anonymity, the privacy level will be determined by the value attributed to the *k* parameter. This value represents the minimum privacy level that the data owners would be willing to admit for their data. Since the privacy level determines the amount of altering required to protect the quasi-identifier attributes, its value will condition the economic value of the medical dataset. It is logical to think that the more restrictive the privacy conditions, the more loss of information the anonymous data will have and, thus, less valuable the data will be. Finally, each data owner will be able to approve or refuse the offer of data interchange (step 4 in Fig. 1). The set of records in the dataset *D* whose owners approved the interchange is ${D}^{{^{\prime}}}=(ID,Q,C)_{i=1}^{m}$, *m* being the number of records in *D*’ such that *m* ≤ *n*. The preferences and conditions of this side of the interactions should not be stored in the blockchain, since they are bilateral interchanges. However, it is possible to use a SSI ledger, such as Hyperledger Indy, for the sole purpose of verifying credentials that contain authorizations of patients to share some given conditions. An emerging good practice for that is using P2P secure communications initiated by the patient using the support of technologies as Aries, combined with a privacy-regulation aware metadata schema, as the Consent Receipt Specification of the Kantara initiative (https://kantarainitiative.org/download/7902/).

### Medical dataset de-identification

The data custodian subjects the medical dataset to a de-identification process (step 5 in Fig. 1) to minimize the patients’ identity disclosure risk and, consequently, the possibility of gaining sensitive information about a specific patient when the dataset is released. The anonymized data should not only meet the owners’ privacy requirements but should also meet the analytical needs of the data customers. Therefore, efforts at making data as useful as possible should be considered during the de-identification process. To achieve these two objectives (privacy and utility preservation), a priori contradictory, we propose that the de-identification process of the medical dataset to be conducted through PPDP methods.

**Figure 5:**
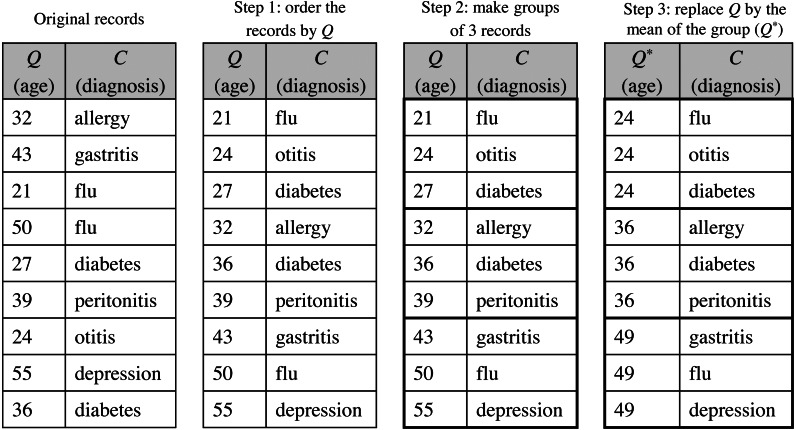
Example of the microaggregation process for *k*= 3 on the quasi-identifier attribute *age* in a dataset of 9 records.

The de-identified version of the dataset *D*′, named *D*^′∗^, follows the privacy requirements established by the patients. ${D}^{{^{\prime}}\ast }=({Q}^{\ast },C)_{i=1}^{m}$ is a modified version of the original dataset, where the direct identifiers have been removed and the quasi-identifiers have been anonymized satisfying a specific protection model, as those discussed in Section ‘Background on data privacy’. Because the ultimate motivation underlying to data disclosure is to conduct analyses on such data, anonymization should be done in a way that the protected data still retain as much analytical utility as possible; that is, the conclusions or inferences extracted from the analysis of the anonymized dataset should be similar to those of the original dataset. For this purpose, the de-identification process will be carried out through the statistical de-identification method using any PPDP method proposed in the literature that satisfies the data protection model stipulated. As an example, Fig. 5 shows the microaggregation process to achieve a *3*-anonymized dataset, where, for simplicity, the quasi-identifier is composed of a single attribute.

To achieve a stronger protection, additional protection models can be applied to confidential attributes, such as discussed in Section ‘Background on data privacy’. In this case, the de-identified dataset will have both quasi-identifiers and confidential attributes anonymized, i.e., ${D}^{{^{\prime}}\ast }=({Q}^{\ast },{C}^{\ast })_{i=1}^{m}$.

### De-identified dataset interchange

Data custodians have the dataset bids available, and may compile de-identified datasets that match both the requirements expressed by the customers and also the preferences of data subjects regarding risk of exposure. That match would result in an offer for a given bid, that the bidder may subsequently finalize, i.e., accept, liberating the payment. That payment may or not be the same amount expressed in the bid, since it is also reflecting the value associated with the preferences of patients. Bids and offers are signals from both sides of the transaction that are necessary to create a self-adjusting price mechanism.

**Figure 6:**
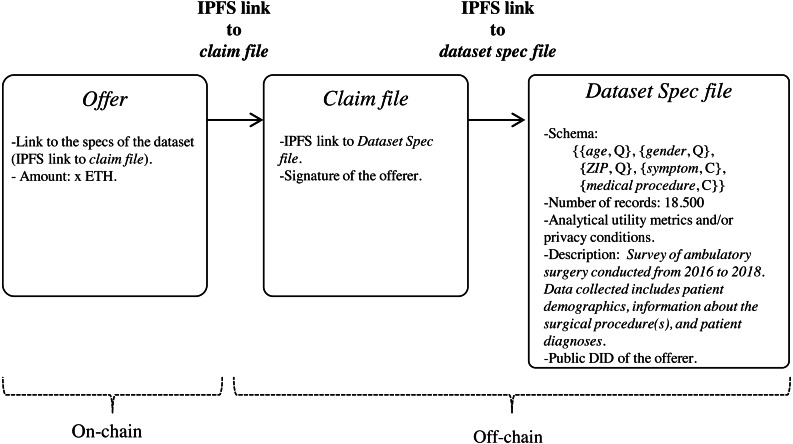
Sequence of IPFS links that must be followed to access the specs of the offered dataset.

Data custodians can response to an (active) bid of interest with an offer (step 6 in Fig. 1). Basically, an offer contains the offered *dataset specs* and the *price*, that is, the amount that the custodian is willing to receive for the de-identified dataset. As in the medical data requirement phase, both the specs and the associated claim are stored in IPFS, with only the IPFS hash of the claim being recorded on the blockchain. As shown in Fig. 6, the specs, in addition to informing about the characteristics of the offered dataset, also contain the custodian’s public DID. This DID will allow the customer to get the custodian’s service endpoint necessary to initiate a secure P2P communication if the offer is accepted. The offer is published on the blockchain through the function offer of the smart contract DatasetBidRegistry. If, after consulting the offer (step 7 in Fig. 1), it is accepted by the customer (step 8 in Fig. 1), the interchange process is initiated.

The interchange agreement is carried out via a trusted digital transaction. Conducting trusted digital transactions involves ensuring the identity of the contracting parties. For this purpose, our model integrates a self-sovereign identity management system to enable verification of the identities of the contracting parties. To prove identity, the contracting parties, i.e., the custodian and the customer, generate verifiable presentations of their credentials as identity proofs, which are interchanged off-ledger via secure P2P communication channels between the agents of their respective wallets (step 8c in Fig. 1) to then be verified on the SSI ledger (step 8d in Fig. 1). The secure P2P communication channel is established in a four-way handshake (step 8b in Fig. 1) initiated by the entity concerned in the offer, that is, the data customer. After verifying the identities, the de-identified medical dataset *D*^′∗^ is shared with the customer (step 8e in Fig. 1) and the amount to be paid for the dataset is transferred to the custodian (step 8 in Fig. 1).

The sequence of steps carried out in the interchange phase of our model is detailed as follows:

1. Connection Invitation (message 1 of the four-way handshake): the agent of the entity concerned in the offer (denoted by *A*) sends a connection invitation message to the agent of the entity that issued the offer (denoted by *B*). Basically, the invitation is a JSON data structure with a session identifier, a public key *PK*_*I*_, and a service endpoint *SE*_*I*_. The message is sent as cleartext to the service endpoint of *B*, which is obtained by resolving on the SSI ledger the public DID of *B* (step 8a in Fig. 1).
2. Connection Request (message 2 of the four-way handshake): the agent of *B* sends a connection request message to the service endpoint *SE*_*I*_ designated in the invitation. The message is encrypted with *PK*_*I*_. The connection request is a JSON data structure that contains the information necessary for *A* to establish a secure connection with *B*, that is, a one-time public key, *PK*_*B*_, and a service endpoint, *SE*_*B*_.
3. Connection Response (message 3 of the four-way handshake): the agent of *A* sends a connection response message to the service endpoint *SE*_*B*_. The message is encrypted with *PK*_*B*_. To obtain *PK*_*B*_ and *SE*_*B*_, the agent of *A* has to decipher the received connection request message by using that private key associated with the public key *PK*_*I*_. The connection response message is a JSON data structure that contains the information necessary for *B* to establish a secure connection with *A*, that is, a one-time public key, *PK*_*A*_, and a service endpoint, *SE*_*A*_.
4. Connection Establishment (message 4 of the four-way handshake): when the agent of *B* receives the connection response message, this is deciphered with the private key associated with the public key *PK*_*B*_. As a result, *B* obtains *PK*_*A*_ and *SE*_*A*_. At this point, both entities hold the public keys and service endpoints necessary to maintain a secure P2P communication channel that allows the exchange of private data. To conclude the four-way handshake, and thus establish the connection, the agent of *B* sends an acknowledgement to the agent of *A*.
5. Interchange of identity proofs: both entities securely interchange their identity proofs, which basically consist of a verifiable credential that proves the entity corporation legally exists (e.g., the verifiable credential of the certificate of incorporation of the corporation) and the countersignature of the entity holding the credential. When an identity proof is received, the receiving entity must consult the information stored on the SSI ledger to verify (1) the integrity of the verifiable credential, (2) the authenticity of the credential issuer, (3) the authenticity of the credential owner, and (4) the status of the verifiable credential, i.e., whether the credential has been revoked.
6. Transfer of the anonymized dataset: the custodian sends the de-identified medical dataset *D*^′∗^ to the customer. Since the P2P connection uses asymmetric encryption to protect the interchange of information, we propose that the custodian generates a content-encryption key (i.e., a symmetric encryption key) and shares it with the customer using the active secure communication session to transmit then the dataset through symmetric encryption, more appropriately for bulk data exchange than asymmetric encryption.
7. Payment: the amount to be paid for the dataset is released in favor of the data custodian and the smart contract is finalized by executing the function finalize of the smart contract DatasetBidRegistry.

### Performance evaluation

In this section we evaluate processing capability of the transactions resulting from the execution of the proposed smart contract on a permissioned blockchain network. For this purpose, we deployed a private Ethereum blockchain network with the Geth (https://geth.ethereum.org/) standalone client of the Go Ethereum implementation, and then, thanks to the Hyperledger Caliper (https://github.com/hyperledger/caliper) benchmark framework, we defined and sent workloads of the proposed smart contract to the blockchain to measure performance metrics. Both Geth and Caliper were run on a 2.50 GHz Intel i5-7200U processor with 2 cores and 16 GB RAM.

We considered as performance evaluation metrics those proposed by the Hyperledger Performance and Scale Working Group (2018):

- Transaction latency, defined as the elapsed average time from when a transaction is submitted until it is added to the ledger and confirmed on the blockchain.
- Transaction throughput, defined as the number of valid transactions per second (tps) that are committed by the blockchain.

The workloads were specified for the *register* (which registers a bid for a dataset) and *offer* (which registers an offer in response to a bid) functions for being the only non-payable functions in the smart contract that generate transactions on the blockchain. The *bidinfo* function only performs searches of transactions on the local ledger of the peer receiving the request and, thus, it does not entail transactions on the blockchain requiring the consensus algorithm, and the *finalize* function is a payable function, which is not supported by Caliper. In our experiments we used one benchmark worker to send the scheduled workloads to the blockchain and test performance, and a blockchain with two peers and one bootnode, the role of the bootnode being to provide the addresses of the network peers to the new peers that join the blockchain. The consensus protocol used during the testing is Clique Proof-of-Authority (PoA).

**Figure 7:**
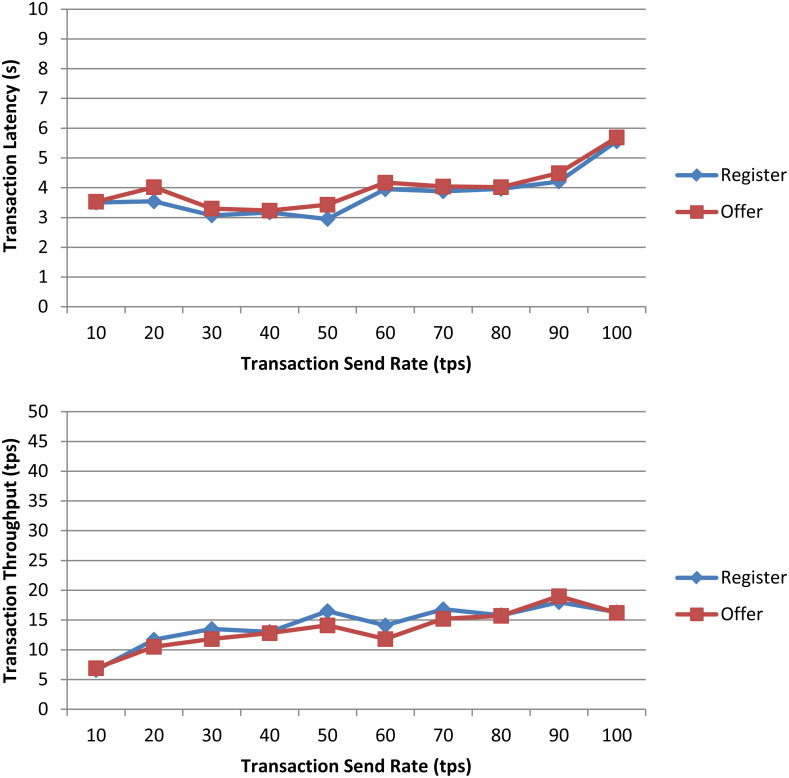
Transaction latency and throughput of the *register* and *offer* functions of the smart contract when the transaction send rate is increased.

**Figure 8:**
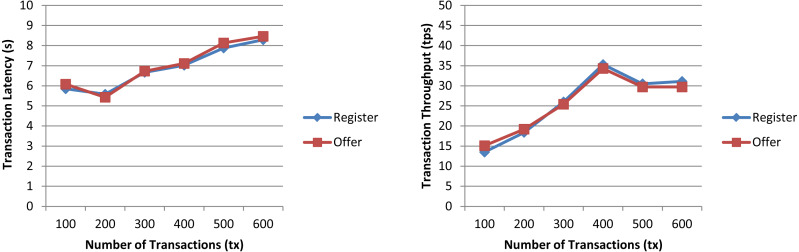
Transaction latency and throughput of the *register* and *offer* functions of the smart contract when the transaction number is increased.

In a first experiment, we measured the transaction latency and throughput for each workload as the number of concurrent requests is increased. First, we performed ten rounds of tests for *register* increasing the transaction send rate by 10 tps until it reached 100 tps, and, then, we repeated the experiment for *offer*. The results depicted in Fig. 7 for both workloads show a good transaction latency (between 3 and 6 s), if we take as a reference point the experiments on the Ethereum blockchain analyzed in the survey of empirical performance evaluation of permissioned blockchain platforms (Dabbagh et al., 2021) (values between 1 and 10 s), and similar transaction throughput, the difference being practically null from 70 tps. In a second experiment, we conducted twelve test rounds (six rounds for *register* and six for *offer*) to measure the performance metrics when the number of transactions to be processed by the blockchain ranges from 100 tx to 600 tx. In all rounds, the send rate was kept at 100 tps, this being the maximum capacity for the nodes used in our experiments. As shown in Fig. 8 and similarly as in (Dabbagh et al., 2021), increasing the number of transactions results in a higher latency, but also in a linear increase throughput, indicating that the smart contract has a reasonable scaling characteristic for both workloads. Similarly as in (Dabbagh et al., 2021), the throughput decreases and flattens out after a certain number of transactions (400 tx in Fig. 8), this being a saturation point determined by the capacity of the nodes used in the test.

## Discussion

The architecture just presented has as a key benefit the property of zero sharing of patient data in the blockchain, not even hashes or links to encrypted files. This is of critical importance, since the immutability (or high cost of mutation) inherent to current blockchain technologies raises concerns on the sharing of such data, even when careful security steps are taken (Finck, 2019; Schwalm, 2020). The use of P2P secure bilateral transfer limits the sharing of data to a maximum while preserving the guarantees of verifiability thanks to verifiable credential technologies as implemented in Hyperledger Indy and Aries. This is in contrast with other proposals in which hashes or links are shared in the blockchain (Azaria et al., 2016).

The resulting design is simple and requires a limited number of specific-purpose components. The complexity of describing the dataset schema is purposefully decoupled from the blockchain. Solutions as archetype descriptions (as in OpenEHR (Haarbrandt, Tute & Marschollek, 2016)) can be used to solve this interoperability issue, thus reusing best practice instead of introducing new conventions that would require eventual mapping.

The central element that makes the design proposed unique is that the data custodian decouples both sides, offer and demand, while fulfilling its usual role as trusted party. That decoupling provides opportunities for different models of operation that bring added benefits. Among them, the following are of special interest:

- Proactively offering particular patients to give consent to sharing. This may be the case, for example, in which customers signal a high demand of some particular kind of data, which may result in an increased economic incentive for users that match the highly demanded profile.
- Reusing dataset sharing by announcing in the blockchain the events of transfer of datasets. This might foster interest and provide additional incentives that require no further anonymization process.
- Identifying customer needs that are in demand but cannot be fulfilled by the patient database available, thus identifying opportunities to aggregate data from different custodians (e.g., networks of healthcare institutions or hospitals).
- To foster EMR adoption among custodians to release secure patients’ information, as well as to empower individuals to make informed decisions about their privacy, as long as they have a finer knowledge and control of the purposes of their own healthcare data. In the long term, it may encourage the sharing of personal health data, without requiring overriding the inalienability of health data in the public interest of, e.g., infectious disease outbreaks.

It should be noted also that the architecture described is auditable. All the transactions at the side patient-custodian are traceable since consent documents are explicit as mandated by current regulations of the GDPR, and the eventual economic transfers are associated to the transactions to the other side. Similarly, the custodian-customer interactions can be audited and matched with the other side of the protocol, in the event of an auditing.

### Conclusions and outlook

Sharing datasets containing health data is critical to secondary use, especially in cases in which data is difficult to find, as in rare diseases. Privacy-preserving data sharing provides the methods and techniques to the sharing of datasets with some levels of anonymity. However, current secure P2P data transfer, verifiable credentials and blockchains can be combined to design more sophisticated mechanisms in which health institutions mediate offer and demand. We have presented the architecture and design of such a solution that features no sharing of patient data beyond bilateral transfer of auditable anonymized datasets, and that allows for tailoring the interchange to the needs and to the preferences of individual patients, for which complete guarantees in terms of personal data rights are observed.

The benefits of using a blockchain setting here include:

- The registering of bids and offers provides transparency and auditability to the process, and the use of DIDs (decentralized identifiers) for the parties involved in the transfer enable the use of P2P secure transfer in a decentralized way. The fact that transfers are committed gets registered immutably in the blockchain, but no patient data is on-chain. This allows for traceability of transfers of datasets, supporting regulatory data protection policies.
- The behaviour of custodians regarding price acceptance or new price proposals provides the required spread of information in the network that is necessary for creating a market, that is driven by demand but reflects the preferences of patients, that are aggregated by custodians.
- The use of hashes and digital signatures for claims and data specs function in a similar way as Ricardian contracts, so that the prose of the specs can be considered part of the commonly agreed commitment between the parties.
- Bids, offers and prices are stored on-chain, but the specs are not, reducing storage needs. Since specs are stored in a decentralized file system, outdated specs might be discarded by the network, or retained only by interested parties.

Future work should be in the direction of the specifics of the implementation. Deployments should account for the setup of the permissioned environment, but this has no differences from a regular deployment of an enterprise blockchain using, for example, Quorum or Hyperledger Besu. The interoperability of schemas should be resolved in the client applications by adopting a common convention that, as mentioned above, could reuse OpenEHR, HL7 models or other existing schemas. The most interesting direction for research, once such a network would be operational, would be related with personal data market dynamics, for which very little is known in the current literature. That would eventually lead to new insights on the values, preferences and behavior of data subjects with regards to health data, and it may be combined with studies in which the way of requesting access is assessed together with the kind of data and perception of risk relative to statistical guarantees of anonymity.

## Additional Information and Declarations

### Data Availability

The following information was supplied regarding data availability: The smart contract code is available at GitHub: https://github.com/msicilia/pp-datasets-sharing
